# Interspecies Variation of *In Vitro* Stability and Metabolic Diversity of YZG-331, a Promising Sedative-Hypnotic Compound

**DOI:** 10.3389/fphar.2017.00527

**Published:** 2017-08-11

**Authors:** Zhihao Liu, Yakun Yang, Li Sheng, Yan Li

**Affiliations:** ^1^Department of Drug Metabolism, Key Laboratory of Active Substances Discovery and Drug Ability Evaluation, State Key Laboratory of Bioactive Substance and Function of Natural Medicines, Institute of Materia Medica, Chinese Academy of Medical Sciences and Peking Union Medical College Beijing, China; ^2^Department of Clinical Pharmacology, College of Pharmacy, Dalian Medical University Dalian, China

**Keywords:** YZG-331, metabolism, CYP450, FMOs, species variation

## Abstract

YZG-331, a synthetic adenosine derivative, express the sedative and hypnotic effects *via* binding to the adenosine receptor. The current study was taken to investigate the metabolic pathway of YZG-331 as well as species-specific differences *in vitro*. YZG-331 was reduced by 14, 11, 6, 46, and 11% within 120 min incubation in human, monkey, dog, rat, and mouse liver microsomes (LMs), respectively. However, YZG-331 was stable in human, monkey, dog, rat, and mouse liver cytoplasm. In addition, YZG-331 was unstable in rat or mouse gut microbiota with more than 50% of prototype drug degraded within 120 min incubation. Interestingly, the systemic exposure of M2 and M3 in rats and mice treated with antibiotics were significantly decreased in the pseudo germ-free group. YZG-331 could be metabolized in rat and human liver under the catalysis of CYP enzymes, and the metabolism showed species variation. In addition, 3 phase I metabolites were identified via hydroxyl (M1), hydrolysis (M2), or hydrolysis/ hydroxyl (M3) pathway. Flavin-containing monooxygenase 1 (FMO1) and FMO3 participated in the conversion of YZG-331 in rat LMs. Nevertheless, YZG-331 expressed stability with recombinant human FMOs, which further confirmed the species variation in the metabolism. Overall, these studies suggested that YZG-331 is not stable in LMs and gut microbiota. CYP450 enzymes and FMOs mediated the metabolism of YZG-331, and the metabolic pathway showed species difference. Special attention must be paid when extrapolating data from other species to humans.

## Introduction

Insomnia is a common problem which has a direct impact on the physical and mental health (Ohayon, 2002). Sedative-hypnotic medication is considered as an important strategy to relieve insomnia (Bertisch et al., 2014). Insomnia medications that are commonly used in clinical treatment mainly focus on the γ-aminobutyric acid receptor, melatonin receptor, orexin, and serotonin site (Zisapel, 2012, 2015). Although effective, they also have some adverse effects including somnolence, dizziness, nausea, headache and hallucinations (Griffiths et al., 1986; Bocca et al., 1999). Based on the complex mechanisms of sleep, several new mechanisms and novel targets have been proposed for treating sleep-wake disorders. To meet divergent medical needs, it is essential to develop novel sedative-hypnotic drugs aiming at new therapeutic targets.

Adenosine is an important endogenous sleep-promoting factor. It not only induces sleep during waking periods, but also exhibits a major role in the modulation of sleep duration or depth (Chen et al., 2013). *Gastrodia elata*, a popular Chinese herbal medicine, has been taken for the therapy of headache, insomnia, dizziness, and so on. N6-(4-hydroxybenzyl)-adenine riboside, an adenosine analog extracted from *Gastrodia elata*, was found to possess potent sedative and hypnotic activity (Huang et al., 2007; Zhang et al., 2012). Subsequently, a detailed structure-effects relationship research of more than 150 derivatives for sedative and hypnotic activity was conducted. Based on the comprehensive evaluation of pharmacodynamic and toxicity data, N6-[(*S*)-1- (phenyl)-propyl]-adenine riboside (YZG-331), was chosen to explore in depth (Liu et al., 2014, 2016).

Previous studies have demonstrated that YZG-331 can shorten the sleep latency, prolong non-rapid eye movement (NREM) sleep time, stabilize the NREM sleep and increase the depth of NREM sleep (patent number: WO2011069294). However, there are certain differences in drug efficacy between mice and rats. For example, sleep electroencephalogram changes were observed in mice treated with 10 mg/kg YZG-331, whereas rats require a higher dose of 50 mg/kg to achieve the similar effect. Since there was no such obvious species difference in sedative and hypnotic activities for other medication as far as we know. For instance, anticonvulsant ED_50_ values of phenobarbital after oral administration in mice and rats were 21.0 and 14.8 mg/kg, respectively. And when administered by subcutaneous injection, there was no significant species difference for diazepam (ED_50_: 0.165 mg/kg in mice, 0.246 mg/kg in rats) and clobazam (ED_50_: 1.24 mg/kg in mice, 0.808 mg/kg in rats) (Shenoy et al., 1982). Therefore, there may be species differences in the metabolism of YZG-331. Despite several adenosine analogs for atrial fibrillation, autoimmune diseases and chronic hepatitis C are in clinical trials (Samsel and Dzierzbicka, 2011), the metabolism studies of adenosine analogs are rare except that Lei et al. (2011) identified major metabolites of N6-(4-hydroxybenzyl) adenine riboside in the plasma and urine of rats.

Metabolism studies play a key role at various stages of drug discovery and development (Kumar and Surapaneni, 2001). In particular, clarification the metabolic pathway and comparison the species differences are vital for prediction of clinical efficacy and species selection for safety studies (Yengi et al., 2007). Besides, the metabolism study of YZG-331 could provide some useful clues to the chemical modifications of adenosine analogs. As far, the research on the metabolism of YZG-331 has not yet been reported. The purposes of the present research were as follows: (1) to identify metabolic pathway of YZG-331 and (2) to compare the species difference in metabolism of YZG-331 *in vitro*.

## Materials and Methods

### Chemicals and Reagents

YZG-331, M2, and YZG-441 (purity ≥ 99%) were obtained from the Department of Natural Products Chemistry (Institute of Materia Medica, Chinese Academy of Medical Sciences). NADPH, NADH, β-NADP, glucose-6-phosphate (6-G-P), glucose-6-phosphate dehydrogenase (6-G-P-DH), midazolam, vanillin, 4-methylumbelliferyl-β-D-glucuronide (4MUG), benzydamine and aminobenzotriazole (ABT) were the products of Sigma Aldrich (St. Louis, MO, United States). Male mouse and rat liver microsomes (LMs)/cytosols were prepared by our group. Pooled mixed-gender human liver microsomes, male beagle dog/monkey liver microsomes, pooled mixed-gender dog/monkey/human liver cytosols, and recombinant human cytochrome P450 enzymes, recombinant human flavin-containing monooxygenases (FMOs) were obtained from BD Gentest (Woburn, MA, United States). HPLC-grade acetonitrile and methanol were purchased from Merck (Darmstadt, Germany). Deionized water was obtained from Milli-Qsystem (Millipore Co., Billerica, MA, United States). All other reagents and solvents were commercially available.

### Animals

Male ICR mice (20–22 g) and Sprague–Dawley rats (220–250 g) were purchased from Vital River Experimental Animal Co., Ltd (Beijing, China). Standard pelleted laboratory chow and water were allowed ad libitum. All experiments were approved by the Animal Care and Use Committee of Peking Union Medical College, and were strictly taken in accordance with Legislation Regarding the Use and Care of Laboratory Animals of China.

### *In Vitro* Metabolism Studies

For the LMs stability study, YZG-331 (0.5 μM) was incubated with human, monkey, dog, rat, and mouse LMs (1 mg/mL) in Tris-HCl buffer (50 mM, pH 7.4, 200 μL) containing MgCl_2_ (5 mM). The medium containing YZG-331 was incubated at 37°C for 5 min, and then NADPH-regenerating system (10 mM β-NADP, 100 mM 6-G-P and 10 U/mL 6-G-P-DH) was added to initial the reaction. The samples were obtained at 15, 30, 45, 60, 90, and 120 min after incubation. All reactions were conducted in triplicate and terminated by adding 400 μL ice-cold acetonitrile. The remaining fraction of YZG-331 was quantified by LC-MS/MS assay. Midazolam (5 μM) was set as the positive control.

For the liver cytosols stability study, human, monkey, dog, rat, and mouse liver cytosols stability study was taken to investigate the metabolic stability of YZG-331. The reaction system contained phosphate buffer (50 mM, pH 7.4, 200 μL) with MgCl_2_ (5 mM), liver cytosols protein (0.5 mg/mL) and YZG-331 (0.5 μM). The samples were collected at 30 min after incubation. All reactions were conducted in triplicate, and then ice-cold acetonitrile (400 μL) was added to terminate the reaction. The remaining fraction of YZG-331 was quantified by LC-MS/MS assay. Vanillin (1 μM), the typical substrate of aldehyde oxidase (AOX), was set as the positive control in the reaction.

For the gut microbiota stability study, the method was taken as described previously (Feng et al., 2015). In short, fresh feces (obtained from six rats/mice) were added into 100 mL anaerobic medium. After mixed thoroughly, the medium was firstly incubated under an anaerobic environment containing N2 atmosphere. Ten microliters of YZG-331 was added into the medium (500 μL) to obtain the final concentration of 5 μg/mL. The cultures were incubated at 37°C. All reactions were conducted in triplicate and terminated by acetonitrile at designated time points (0, 15, 30, 60, 120 min). The remaining fraction of YZG-331 was quantified by LC-MS/MS assay.

Intestinal bacteria from pseudo germ-free (PGF) rats and mice were used to further investigate the role of intestinal bacteria in the metabolism of YZG-331. Briefly, male rats or mice were orally given cefadroxil (100 mg/kg) twice a day for 3 days to reduce gastrointestinal bacteria levels, and the colon contents of the animals were harvested after the last treatment of antibiotics. Furthermore, 4MUG (5 μg/mL), a substrate of intestinal bacterial β-glucuronidase, were used as the positive control for the fecal incubation system.

### Identifications of YZG-331 Metabolites

To identify metabolites, YZG-331 (10 μM) was incubated with LMs (human, monkey, dog, rat, mouse) or gut microbiota as described above. In addition, the incubation of YZG-331 with gut microbiota was terminated by high temperature inactivation (90°C, 5 min). Then, the supernatant samples were continued to incubate with LMs. Briefly, the mixed medium contained phosphate buffer (50 mM, pH 7.4) with MgCl_2_ (5 mM), LMs protein (0.5 mg/mL), NADPH-regenerating system and supernatant of gut microbiota incubation (10 μL) in a total volume of 200 μL. The incubation was terminated by acetonitrile after 30 min and centrifuged at 14,000 rpm for 5 min. Then the samples were analyzed by LC-MS/MS.

### Identification of Enzymes Responsible for the Hydroxylation of YZG-331 in LMs

The contributions of CYP450s and FMOs to the metabolism of YZG-331 and M2 in rat LMs were investigated by using a nonspecific CYPs inhibitor ABT and thermal inactivation of FMOs. YZG-331/M2 (0.5 μM) was incubated with rat LMs (1 mg protein/mL) with or without ABT (1 mM) for 30 min and the reaction was stopped with 2 volumes of acetonitrile. Thermal inactivation of FMOs was carried out by preheating rat LMs (1 mg protein/mL) at 50°C for 5 min. Then rat LMs was immersed in ice-cold bath followed by addition of YZG-331/M2 (0.5 μM). After a 3 min equilibration, the reaction was started by NADPH. The incubation was stopped after 30 min by adding 2 volumes of acetonitrile. The mixtures were centrifuged at 14,000 rpm at 4°C for 5 min, and supernatant samples were determined using LC-MS/MS. In this study, benzydamine (20 μM), a known substrate of FMO, was used as the positive control for FMO activity. The retained activity of CYPs was confirmed by using midazolam (a CYP3A substrate). The remaining fraction of YZG-331 and metabolites of control drugs were analyzed using LC-MS/MS.

Human cDNA-expressed CYPs were used to investigate the enzymes mediated the metabolism of YZG-331. YZG-331 (0.5, 2, and 5 μM) and 20 pmol of thirteen individual human cDNA expressed CYPs (CYP1A1, 1A2, 2B6, 2C8, 2C9, 2C19, 2D6, 2E1, 2J2, 3A4, 4A11, 4F2, and 4F3) and three cDNA expressed FMO enzymes (50 pmol/mL FMO1, FMO3, and FMO5) under the conditions similar to those of rat LMs (described above), except incubating time (120 min). Probe substrates of CYPs were used as the positive controls, and the incubation without NADPH served as the negative controls.

### *In Vivo* Studies in PGF Rats and Mice

Male rats or mice were orally treated with cefadroxil (100 mg/kg) for 3 days as described above, then *in vivo* study was taken 2 days after the antibiotic treatment. The rats were oral received a single dose of YZG-331(50 mg/kg for rats, 20 mg/kg for mice). Then blood was harvested immediately through posterior orbital venous plexus at 0, 0.08, 0.25, 0.5, 1, 2, 4, 8, and 24 h post-YZG-331 treatment. Plasma (100 μL for rats, 10 μL for mice) was immediately centrifuged at 5000 rpm for 10 min. The samples of plasma from rats or mice were prepared as describe before (Liu et al., 2014, 2016).

### LC-MS/MS Determination

The samples were quantified by HPLC-MS/MS consisted of mass spectrometer (API4000; Applied Biosystems, USA), HPLC system (1260; Agilent, United States) and Zorbax SB-C_18_ column (3.5 mm, 2.1 mm × 100 mm, Agilent, Santa Clara, CA, United States). An electrospray ionization (ESI) source was used in positive mode. The mobile phase for YZG-331, Midazolam, 4-MUG and benzydamine consisted of acetonitrile/water (0.1% formic acid). The mobile phase for vanillin consisted of solvent A (1 mM ammonium acetate in methanol) and solvent B (0.05% ammonia in water). The ionization was conducted using a positive ion mode for YZG-331, Midazolam, benzydamine, and 4MUG and in negative ion mode for vanillin. The selected transitions of m/z were m/z 386.2→254.2 for YZG-331,338.7→291.3 for midazolam, 310.0→264.9 for benzydamine, m/z 166.2→151.8 for vanillin and 338.7→177.4 for 4MUG. The flow rate was 0.2 mL/min and the operating temperature was 25°C for all determinations.

For the metabolites detection, full-scan MS spectra was used to measure the masses of YZG-331 and its metabolites. The mass spectrometer was selected in the positive ion mode in the mass range of m/z 100-800. The MS/MS spectra were produced by collision-induce dissociation of the selected precursor ions. The mobile phase consisted of water (A)/acetonitrile (B) (0.1% formic acid) with a flow rate of 0.2 mL/min. The following condition was used: 90% A at 0-6 min, 90-10% A at 6–15 min, 10–90% A at 15–25 min. Then, the starting conditions were reestablished and balanced for 2 min. The sample injection volume was 10 μL. The parameters were selected to get maximum sensitivity at the unit resolution.

### Data Analysis

The pharmacokinetic parameters for YZG-331/M2 in plasma were determined by noncompartmental analysis using Phoenix WinNonlin 6.3 (Pharsight Corporation, Mountain View, CA, United States). Statistical analyses between the two groups were performed with a two-tailed Student’s *t*-test, while multi-comparisons were performed using one-way ANOVA. Values of *p* < 0.05 were considered to statistically significant. The contents of metabolites from LMs incubation were evaluated by principal component analysis (PCA) using SPSS software (Li et al., 2016; Ma et al., 2016).

## Results

### Metabolism of YZG-331 in Liver Microsomal/Cytosolic Fractions and Gut Microbiota

**FIGURE 1:**
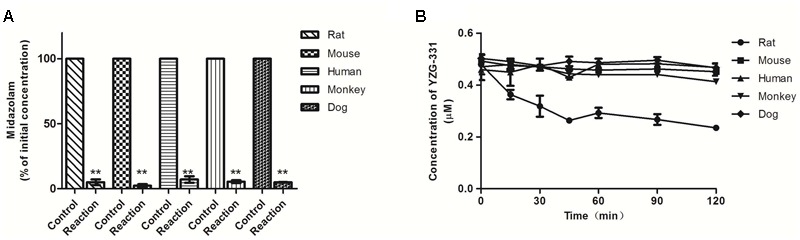
Stability of YZG-331 in liver microsomes (LMs) of human, dog, monkey, mouse, and rat *in vitro*. Positive control midazolam (0.5 μM, **A**) and YZG-331 (0.5 μM, **B**) was incubated at 37°C for 120 min in for all species. The control groups for **(A)** represent the content of midazolam or YZG-331 in the inactivate micosomes. The reaction groups for **(A)** represent the remain content of midazolam after incubating with NADPH at 37°C for 30 min. **(B)** Represents the remain content of YZG-331 after incubating with NADPH at 37°C for 120 min. Each column is the mean ± SD of triplicate determinations. Statistical differences of each point were compared with those for the control groups by a two-tailed unpaired *t*-test, with *p* < 0.05 as the limit of significance (^∗∗^*p* < 0.01 vs control; ^∗^*p* < 0.05 vs. control).

**FIGURE 2:**
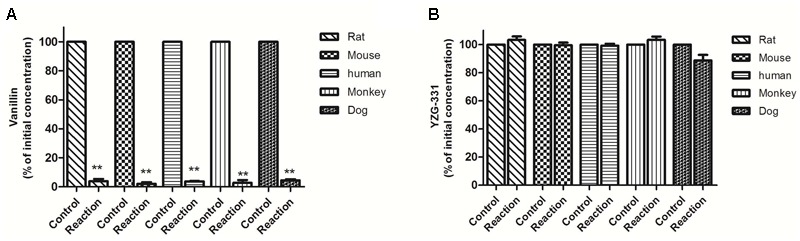
Stability of YZG-331 in liver cytosol of human, dog, monkey, mouse, and rat *in vitro*. **(A)** The positive control vanillin (1 μM) incubated at 37°C for 30 min in liver cytosol for all species; **(B)** YZG-331 (0.5 μM) was incubated at 37°C for 30 min in liver cytosol for all species. The control groups represent the content of vanillin or YZG-331 in the inactivate liver cytosol. Each column is the mean ± SD of triplicate determinations. Statistical differences of each point were compared with those for the control groups by a two-tailed unpaired *t*-test, with *p* < 0.05 as the limit of significance (^∗∗^*p* < 0.01 vs. control; ^∗^*p* < 0.05 vs. control).

As preliminary identification of the possible metabolites and metabolic pathway, incubations were taken in human, monkey, dog, rat, and mouse LMs and cytosolic fractions with YZG-331 as the substrate. The results were shown in **Figure 1**. The positive control midazolam, a substrate of CYPs, was decreased by more than 90% in the presence of NADPH in all species of LMs, indicating the LM systems was reliable. Approximately 46% of YZG-331 was metabolized in rat LMs in the presence of NADPH. Less than 20% of YZG-331 was diminished in human, monkey, dog, and mouse LM systems. In addition, the positive control vanillin, a substrate of AOX, was reduced in human liver cytosol (**Figure 2**). However, YZG-331 was stable in all species of cytosol, which suggested that cytosolic enzymes are not involved in the metabolism of YZG-331.

**FIGURE 3:**
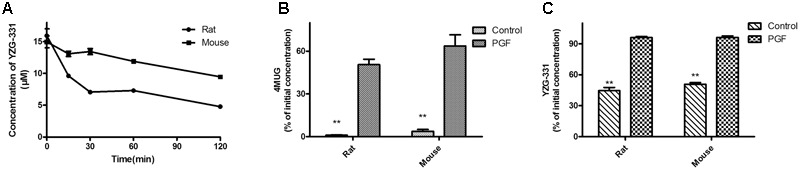
Stability of YZG-331 in gut microbiota from normal or pseudo germ-free (PGF) animals. **(A)** YZG-331 (5 μg/mL) was added into the fresh rats or mice intestinal bacteria cultures. The cultures were incubated at 37°C for 120 min; **(B)** male rats or mice were orally treated with cefadroxil (100 mg/kg) twice a day for 3 days, and the colon contents of the animals were collected on the third days after the final treatment with antibiotics. 4MUG (5 μg/mL), as the positive control, was added into the intestinal bacteria cultures and incubated at 37°C for 120 min. **(C)** YZG-331(5 μg/mL) was added into the intestinal bacteria cultures from PGF animals and incubated at 37°C for 120 min. Each column is the mean ± SD of triplicate determinations. Statistical differences of each point were compared with those for the control groups by a two-tailed unpaired *t*-test, with *p* < 0.05 as the limit of significance (^∗∗^*p* < 0.01 vs. PGF).

Furthermore, we investigated whether gut microbiota is responsible for the biotransformation of YZG-331. Our data showed that YZG-331 was unstable in rat or mouse gut microbiota with more than 50% of prototype drug degraded in 120 min (**Figure 3A**). On the other hand, gut microbiota obtained from PGF rats or mice was taken to confirm that gut microbiota was responsible for the conversion of YZG-331. As shown in **Figure 3B**, antibiotics treatment inhibited the bacterial mediated metabolism of 4MUG, suggesting an intestinal bacteria scarcity environment in the animals. As expected, the lack of intestinal bacteria resulted in a low level of metabolism of YZG-331 (**Figure 3C**), implying that gut microbiota can act as an “organ” that involved in the conversion of YZG-331. Subsequently, metabolite identification following incubation was conducted.

### Identification of Metabolites of YZG-331 *In Vitro*

**FIGURE 4:**
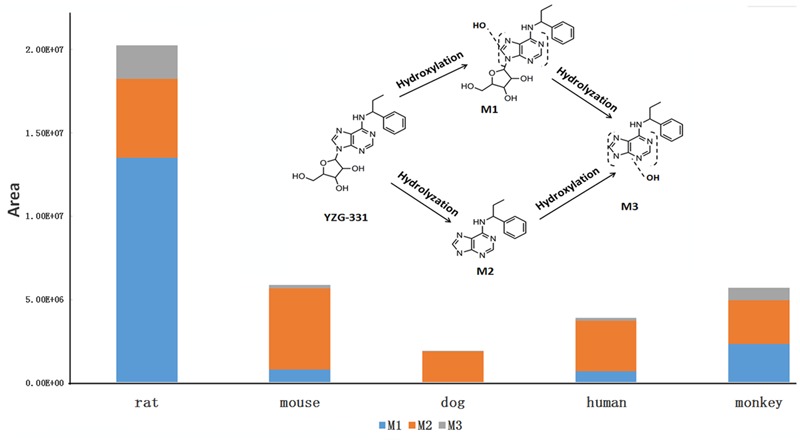
The detailed structures and contents of YZG-331 metabolites in LMs. YZG-331 (10 μM) was incubated at 37°C for 120 min for all species. Due to the scarcity of standards, the amount of metabolites was determined by peak area ratio of metabolite to internal standard.

**FIGURE 5:**
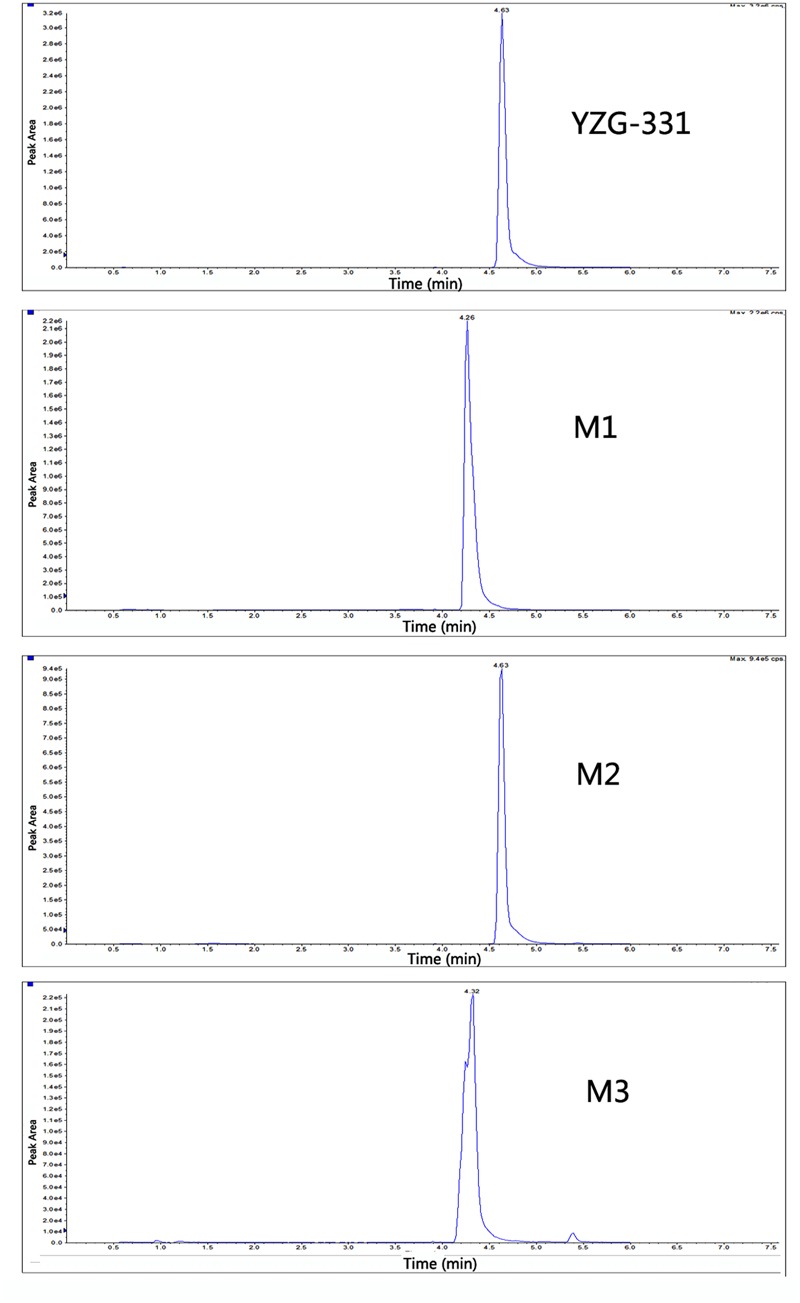
The extracted ion chromatograms of YZG-331 metabolites in rat LMs. YZG-331 (10 μM) was incubated at 37°C for 120 min for all species. The MS/MS spectra was acquired for the accurate measurement of the masses of YZG-331 and its metabolites. The figure only expressed the chromatograms from rat liver microsomes (LMs) were selected as the representative.

**FIGURE 6:**
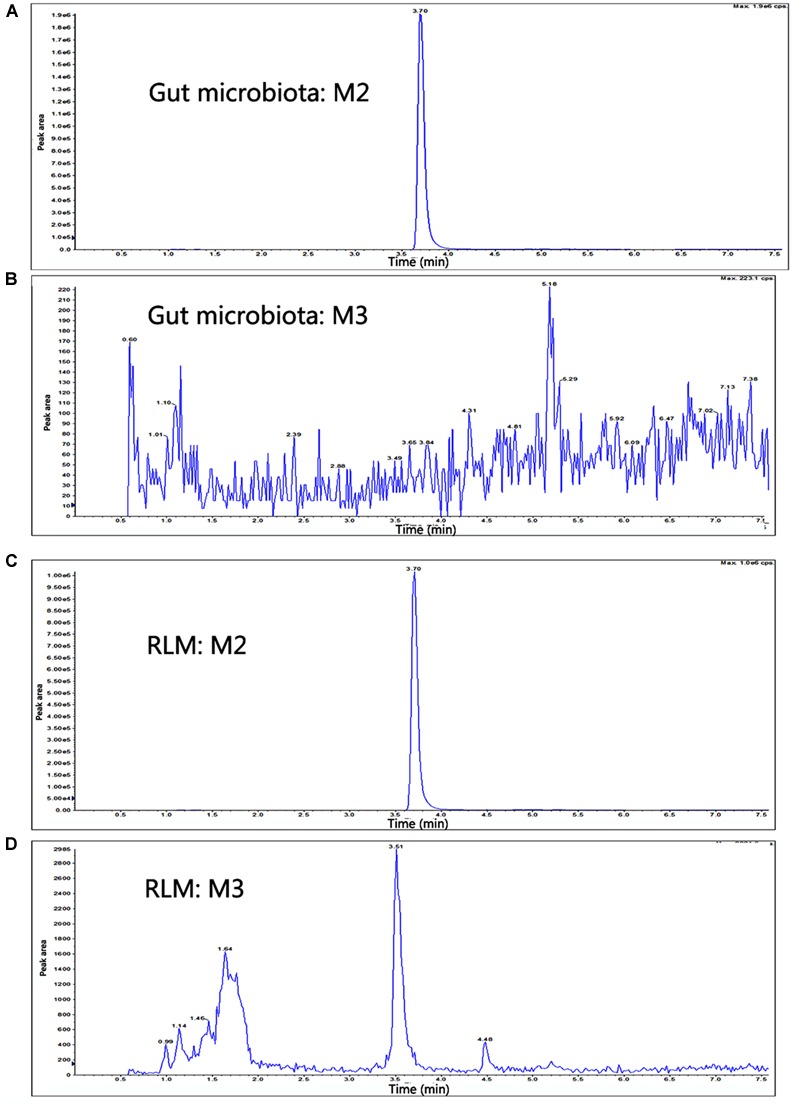
The extracted ion chromatograms of M2 and M3 in gut microbiota or LMs system from supernatant of gut microbiota. **(A,B)** The gut microbiota cultures of YZG-331 (10 μM) were incubated at 37°C for 120 min. **(C,D)** The supernatant obtained from the gut microbiota incubation was continued to incubate with rat LMs at 37°C for 120 min. The MS/MS spectra was acquired for the accurate measurement of the masses of YZG-331 and its metabolites.

In total, three metabolites were identified from the incubated YZG-331 in the LMs of five different species using LC-MS/MS (**Figures 4, 5**). Among these, the molecular of M1 (*m/z* 402) was showed 16 Da higher than that of YZG-331, indicating that it would be the hydroxylation of parent drug. M2 (*m/z* 254) was the hydrolysis product of parent drug at the site of N9 terminal of N6-(4-hydroxybenzyl) adenine through the C–N bond. The molecular of M3 (*m/z* 270) was 16 Da higher than that of M2, indicating that it would be hydroxylation metabolite of M2 or hydrolysis product of M1. M2 was identified as the major metabolite via gut microbiota conversion (**Figure 6**), suggesting hydrolyzation was the critical pathway of the parent compound in gut microbiota. Interestingly, M3 was detected when the supernatant of gut microbiota was incubated in the LMs system, confirming that M3 was the hydroxyl style of M2. The detailed information, structures, contents of metabolites and total ion chromatograms of YZG-331 metabolites are shown in **Table 1**. Compared with other species, metabolite content in rat LMs is relatively high (**Figure 4**).

**Table 1:** Metabolic reaction, nominal mass shift, and MS/MS fragments of YZG-331 and its metabolites.

Metabolites	Metabolic reaction	[M+H]^+^	Nominal mass shift	MS/MS fragments
YZG-331		386	0	254, 136, 119
M1	Hydroxylation	402	+16	270, 136
M2	Hydrolyzation	254	-132	136, 119
M3	Hydrolyzation+ Hydroxylation	270	-132+16	136, 152

### *In Vivo* Pharmacokinetics of YZG-331 in PGF Rats and Mice

**FIGURE 7:**
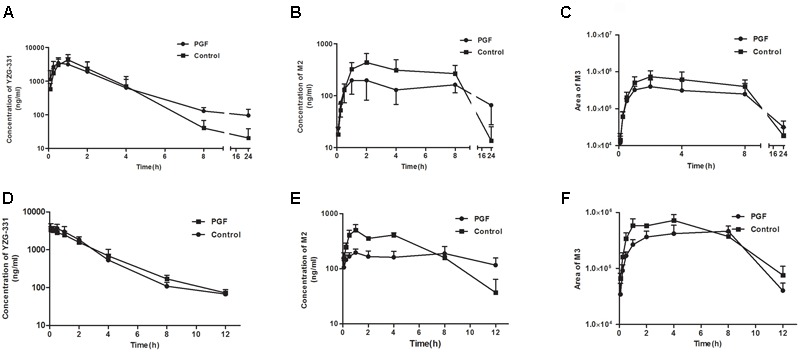
*In vivo* pharmacokinetics of YZG-331 in PGF rats and mice. Plasma concentration of YZG-331 **(A,D)**, M2 **(B,C)**, and M3 **(E,F)** in rats and mice, respectively. Male rats or mice were orally treated with cefadroxil (100 mg/kg) twice a day for 3 days, and pharmacokinetics was performed 2 days after final administration. A single dose of YZG-331(50 mg/kg for rats, 20 mg/kg for mice) was given through oral administration. Each column is the mean ± SD of five determinations.

As shown in **Figures 7A,D**, absorption of YZG-331 was rapid after oral administration. The plasma concentrations of YZG-331 were maximal at 0.25 h in mice and within 2 h in rats. The mean peak concentration (*C*_max_) of YZG-331 in mice after a single oral administration at 20 mg/kg was 4.07 μg/mL, whereas *C*_max_ was 4.4 μg/mL in rats at a dose of 50 mg/kg. The mean residence times (MRT) were 2.63 ± 0.84 h and 3.45 ± 0.29 h in rats and mice, respectively. To investigate the role of gut microbiota in the pharmacokinetics of YZG-331, we used PGF rats and mice in the study. As shown in **Figure 7** and **Table 2**, the systemic exposure of YZG-331 was not changed obviously in rats and mice with antibiotics compared with non-antibiotic group. However, the *C*_max_ and AUC values of M2 and M3 in rats and mice treated with antibiotics were significantly decreased in PGF group (*p* < 0.05, **Figure 7** and **Table 3**). The results suggested that the antibiotic-induced decrease in intestinal bacteria resulted in a low level of conversion of YZG-331 into M2 in the intestine and thus a low level of M2 as well as M3 in the blood.

**Table 2:** Pharmacokinetic parameters of YZG-331 in rats and mice after a single oral administration of YZG-331.

Parameter	Unit	Rat^a^		Mouse^b^	
		Control	PGF	Control	PGF
*T*_1/2β_	h	4.14 ± 1.38	5.85 ± 1.56	6.28 ± 3.02	5.26 ± 2.62
*T*_max_	h	1.10 ± 0.55	0.55 ± 0.27	0.25 ± 0.23	0.2 ± 0.18
*C*_max_	ng/mL	4441 ± 1718	3936 ± 1814	4072 ± 663	3620 ± 1185
AUC_(0-t)_	h^∗^ng/mL	11009 ± 4572	11186 ± 6615	10717 ± 1318	10417 ± 2546
AUC_(0-∞)_	h^∗^ng/mL	11155 ± 4462	12048 ± 6426	11338 ± 1206	10936 ± 2450
MRT_(0-t)_	h	2.63 ± 0.84	4.49 ± 1.20	3.45 ± 0.29	3.77 ± 0.92
MRT_(0-∞)_	h	3.24 ± 1.76	6.98 ± 3.31	5.20 ± 1.49	5.19 ± 1.86

*Data shown are the means ± SD, *n* = 5 for each group.*

*^a^50 mg/kg, p.o.; ^b^20 mg/kg, p.o.*

**Table 3:** Pharmacokinetic parameters of M2 and M3 in rats and mice after a single oral administration of YZG-331.

Metabolite	Parameter	Unit	Rat^a^		Mouse^b^	
			Control	PGF	Control	PGF
M2						
	*T*_max_	h	3.00 ± 2.83	3.25 ± 3.20	1.60 ± 1.34	4.00 ± 3.67
	*C*_max_	ng/mL	507 ± 134	224 ± 95^∗∗^	516 ± 112	208 ± 49.9^∗∗∗^
	AUC_(0-t)_	h^∗^ng/mL	4682 ± 601	3053 ± 776^∗∗^	3438 ± 608	2778 ± 455
	AUC_(0-∞)_	h^∗^ng/mL	29332 ± 8538	27948 ± 6318	3654 ± 759	5418 ± 5366
M3						
	*T*_max_	h	3.60 ± 2.61	2.00 ± 1.22	3.60 ± 0.89	7.20 ± 1.79
	*C*_max_	×10^2^area	8142 ± 2515	4092 ± 2243^∗^	7458 ± 1541	5044 ± 1334^∗^
	AUC_(0-∞)_	×10^2^h^∗^area	75709 ± 16298	45834 ± 34993	61685 ± 7373	45654 ± 10444^∗^
	AUC_(0-∞)_	×10^2^h^∗^area	76953 ± 16506	49332 ± 34317	69429 ± 12987	56382 ± 16837

*Data shown are the means ± SD, *n* = 5 for each group. ^a^50 mg/kg, p.o.; ^b^20mg/kg, p.o. ^∗^*P* < 0.05, ^∗∗^*P* < 0.01, ^∗∗∗^*P* < 0.001.*

### Identification of Enzymes Responsible for the Metabolism of YZG-331 in LMs

**FIGURE 8:**
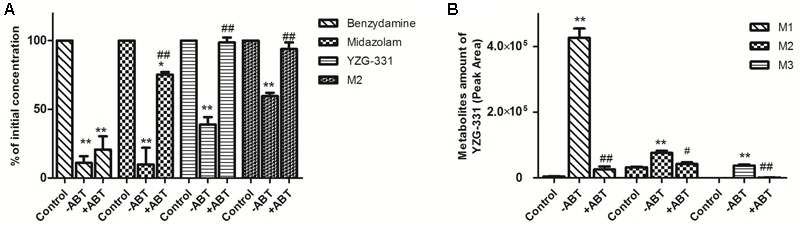
Effects of CYP450 on the metabolism of YZG-331 in rat liver microsomes. **(A)** YZG-331/M2 (0.5 μM) was incubated with rat LMs (1 mg protein/mL) and NADPH-generating system in the absence and presence of ABT (1 mM) at 37°C for 30 min. Midazolam (5 μM) and benzydamine (20 μM) were set as positive and negative control, respectively; **(B)** M1, M2, and M3 production from YZG-331 with or without of ABT. Statistical differences of each point were compared with those for the control groups by a two-tailed unpaired *t*-test, with *p* < 0.05 as the limit of significance (^∗∗^*p* < 0.01 vs. control; ^##^*p* < 0.01 vs group without ABT). Each column is the mean ± SD of triplicate determinations.

Rat LMs was used to further determine whether the CYPs were responsible for the metabolism of M1, M2 and M3. As shown in **Figure 8A**, the CYPs inhibitor ABT could strongly reduce the metabolism of CYP3A4 substrate midazolam but not the negative control benzydamine, which suggested the system was reliable. The metabolism of YZG-331 and M2 was also blocked in the incubation mixtures in the presence of ABT (**Figure 8A**). When YZG-331 was incubated with rat LMs, M1, M2, and M3, were decreased in the presence of ABT (**Figure 8B**). The results clearly showed that CYPs were responsible for the formation of M1, M2, and M3.

**FIGURE 9:**
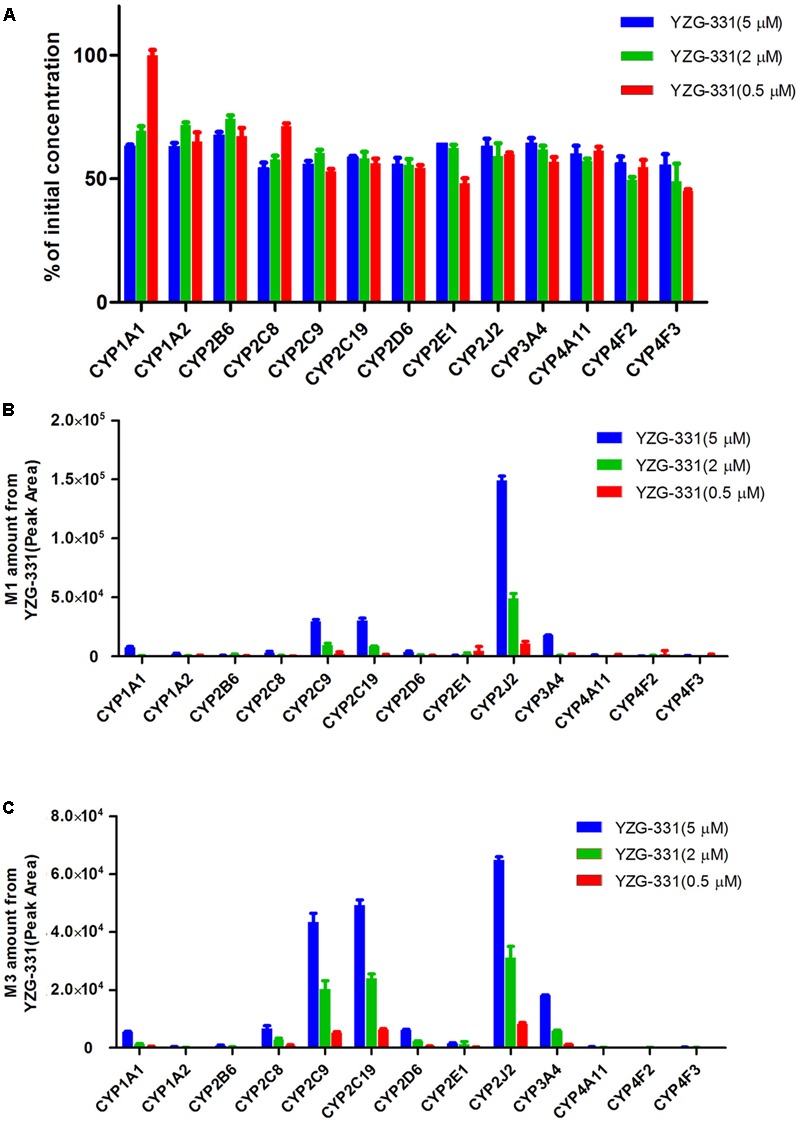
Effects of CYP450 on the metabolism of YZG-331 in human cDNA expressed CYPs system. **(A)** YZG-331 (0.5, 2, and 5 μM) and 20 pmol of thirteen individual human cDNA expressed CYPs (CYP1A1, 1A2, 2B6, 2C8, 2C9, 2C19, 2D6, 2E1, 2J2, 3A4, 4A11, 4F2, and 4F3) were incubated at 37°C for 30 min. **(B,C)** The production of M1 **(B)** and M3 **(C)** from YZG-331 mediated by recombinant human CYPs. Each column is the mean ± SD of triplicate determinations.

Toward a more detailed understanding of the roles of CYP enzymes in the biotransformation of YZG-331, the contribution of 13 recombinant human CYP enzymes in the metabolism of YZG-331 was investigated. As shown in **Figure 9**, the recombinant CYP2J2 showed the highest metabolic activity for the formation of M1 and M3, while the recombinant CYP2C9 and CYP2C19 was involved in the formation of M1 and M3. Moreover, recombinant CYP1A2 and CYP3A4 may also participate in the formation of M1 and M3 to a certain extent.

**FIGURE 10:**
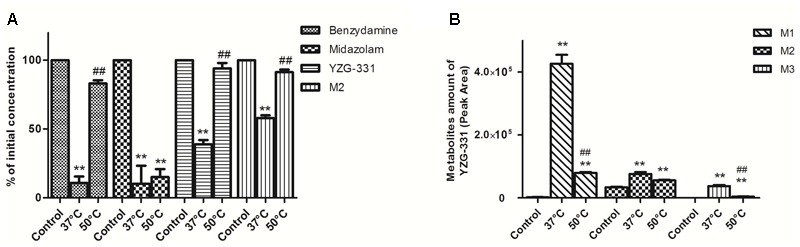
Effects of FMOs on the metabolism of YZG-331 in rat liver microsomes. **(A)** YZG-331/M2 (0.5 μM) was incubated with Rat LMs at 37 or 50°C for 5 min, respectively. Midazolam (5 μM) and benzydamine (20 μM) were set as negative and positive control, respectively; **(B)** Production of M1, M2, and M3 from YZG-331was detected when YZG-331 was incubated at 37 or 50°C, respectively. Each column is the mean ± SD of triplicate determinations.

To distinguish between the contributions of FMOs and CYPs to the metabolism, incubations can be carried out on microsomes that have been preheated at 50°C in the absence of NADPH, which knocks out FMOs activity. As expect, we found that the metabolism of benzydamine (known substrate of FMOs) other than midazolam (known substrate of CYP3A4) was strongly decreased at the condition 50°C (**Figure 10A**), which suggested that the activity of FMOs was successfully knocked out. In our study, the metabolism of YZG-331 was effectively inhibited by the thermal inactivation of FMOs, resulting in a 30% reduction in M2 formation and 50–60% decrease in the formation of M1 and M3 (**Figure 10B**). Thus, FMOs may also play a role in YZG-331 metabolism

**FIGURE 11:**
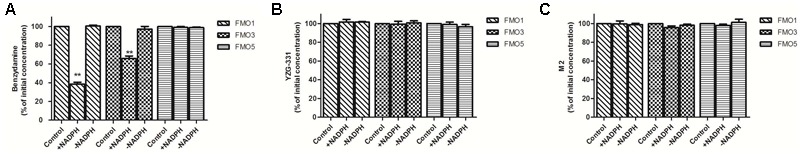
Effects of FMOs on the metabolism of YZG-331 in recombinant human FMOs system. **(A)** Positive control benzydamine (20 μM) was incubated at 37°C for 120 min in recombinant human FMO1, FMO3, and FMO5, respectively; **(B,C)** YZG-331/M2 (0.5 μM) was incubated at 37°C for 120 min in recombinant human FMO1, FMO3, and FMO5, respectively. Each column is the mean ± SD of triplicate determinations.

To further confirm the role of human FMOs in the metabolism of YZG-331, we examined the metabolism of YZG-331/M2 with recombinant human FMOs. As shown in **Figure 11A**, benzydamine was not stable in hFMO1 and hFMO3 but not hFMO5 in the presence of NADPH. Probably because that FMO5 displays no or poor reactivity toward classical FMO substrates and, consequently, was believed not to play a significant role in drug conversion (Phillips and Shephard, 2017). However, no metabolites of YZG-331 were observed in the incubations with recombinant FMO1, FMO3, and FMO5 (**Figures 11B,C**).

### Comparison of YZG-331 Metabolism among Different Species

**FIGURE 12:**
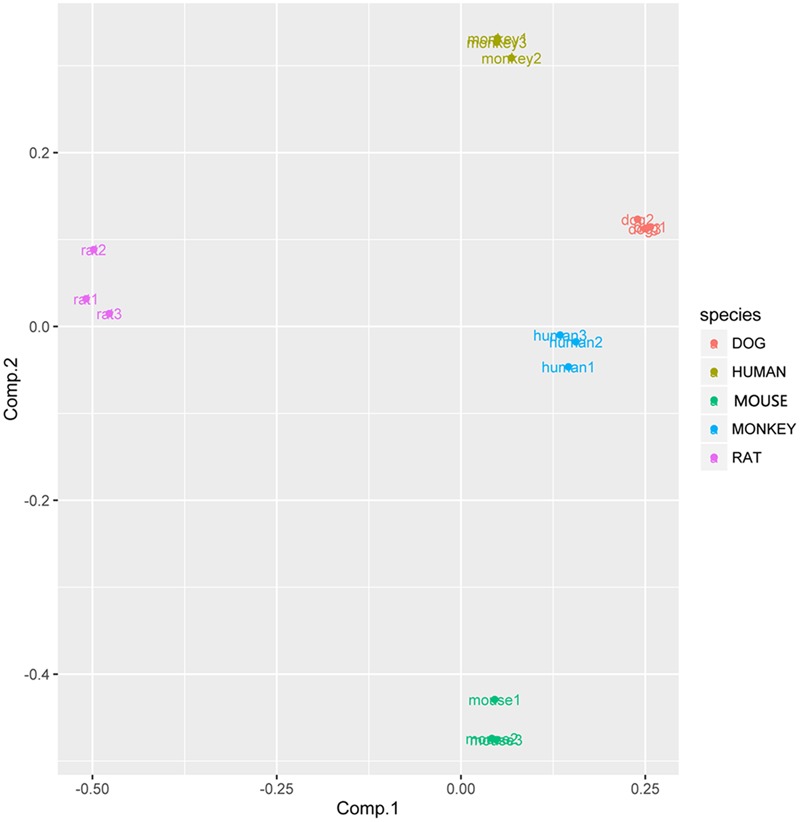
PC score plots for metabolism profiles of YZG-331 in LMs from five species. YZG-331 (10 μM) was incubated at 37°C for 120 min for all species. The content of the metabolites from 120 min incubation was selected to take the PCA analysis.

In view of the metabolism data, PCA was conducted during different species. The PCA score scatter plots of rat LMs expressed distinctive and discrete clusters for other species (**Figure 12**), implying a interspecies difference for YZG-331 metabolism. According to the PCA results, the human groups were clustered with dog groups. Furthermore, the monkey groups and dog groups cluster together.

## Discussion

Drug metabolism research is an indispensable part at various stages of drug discovery and development (Yan and Caldwell, 2001). YZG-331 is an attractive sedative hypnotic candidate. A comprehensive investigation into the metabolic pathways involved in the clearance of YZG-331 can reveal the pharmacological activities and toxicological implications to ensure safe and effective use of the drug (Xiao et al., 2012).

It is well accepted that intestinal bacteria has an irreplaceable role in the conversion of orally administrated drugs (Yao et al., 2016). Before being absorbed through the intestinal barrier, drugs may be transformed by intestinal bacteria leading to the occurrence of more active, toxic or ineffective compounds. This course is vital for whether drug candidates will become therapeutics. As a promising sedative-hypnotic drug, YZG-331 would be administered orally and inevitably lead to contact with intestinal microflora in the gastro-intestinal tract and subsequently be transformed by intestinal bacteria before being absorbed into the blood. YZG-331 could be firstly converted to its hydrolysis form (M2) by the majority of the isolated intestinal bacteria (**Figures 3, 6**). This study presents an interesting case to show the importance of the gut microbiota in modifying M2 systemic exposure. Oral administration of antibiotics decreased the amount of intestinal bacteria and thus suppressed M2 generation, resulting in a low level of M2 in the blood (**Figure 7**). This study raises an important issue for patients orally receiving drug treatment together with antibiotics may be modified the converting of YZG-331 through antibiotic-mediated changes in the gut microbiota. In the study, we found that, compared with M1 and M3, the generation of M2 was not significantly influenced by ABT in rat LMs. The results indicated that M2 was not mainly converted by microsome enzymes. Thereby, the hydrolysis product M2 could be mostly generated by gut microbiota. In contrast, the hydroxyl products M1 and M3 could by generated by the enzymes expressed in the liver. To sum up the evidence, we deduce that YZG-331 was firstly, at least in part, converted to M2 by the intestinal bacteria, and M3 was the hydroxyl style of M2.

Previous studies have shown that there were some differences in the efficacy of YZG-331 between mice and rats. Generally, rats require a higher dose to achieve similar efficacy to mice. Correspondingly, the *C*_max_ of YZG-331 in mice after oral administration of 20 mg/kg was comparable to that of rat at a dose of 50 mg/kg. The metabolic stability study of drugs in LMs helps to assess the contribution of the first pass effect to bioavailability. In the present study, the metabolism of YZG-331 was thoroughly investigated using liver microsomes/cytosol from different species *in vitro*. Interestingly, significant variations have been observed between species in the current study. YZG-331 was found to be metabolized faster in rat LMs than in other four species (**Figure 1**). AOX and xanthine oxidase are two important cytosolic enzymes (Sperling, 2012). They participate in the metabolism of some important purine related drugs, such as azathioprine and vidarabine. However, YZG-331 was relatively stable in the liver cytoplasm of all the species (**Figure 2**), which suggested microsomes would be the target site for the metabolism and species difference. The large differences in the elimination of YZG-331 suggested that the interspecies extrapolation from animals to human should be aware.

CYPs, which constitute a heme-containing superfamily, are responsible for the bulk of the oxidative metabolism of known drugs in humans. In this study, we identified the enzymes responsible for the oxidative metabolism of YZG-331. As expected, multiple CYPs were found to be involved in the oxidative metabolism of YZG-331 by recombinant enzymes. In the study, YZG-331 is confirmed to be metabolized by rat CYPs to yield a hydroxyl product, M1, as the main circulating metabolite (**Figures 4, 8**). Several human CYPs consistently metabolized the YZG-331 screened in the present study, which revealed that YZG-331 would be widely metabolized *in vivo* and probably affected by many factors. Among them, CYP2C8, 2C9, 2C19, CYP2J2, and CYP3A4-mediated formation of M1 and M3, indicates that YZG-331 mainly undergoes hydroxylation reaction in liver (**Figures 9B,C**). Apart from CYP3A and 2C family, CYP2J2 belongs to a relatively newer CYP subfamily (Karkhanis et al., 2017). Intriguingly, CYP2J2 attained important role of M1 and M3 production in human LMs, even with low concentrations of YZG-331. We postulated there was relative high affinity between CYP2J2 and YZG-331, resulting in the considerable production of M1 and M3 in human LMs. This multiple metabolism pathway would decrease the potential of risk in the application of YZG-331 in the clinic.

According to the results, the stability of YZG-331 was remarkably varied in different species, and it could be partly related to CYP differences among species. Although CYPs are highly conserved among species, there are still some variation in the amino acid sequences (Martignoni et al., 2006). However, even small changes in the amino acid sequences could lead to large variation in substrate selectivity and catalytic activity. Thus, differences in CYPs could result profound variations in drug metabolism (Guengerich, 1997). In the study, it was suggested that YZG-331 was not stable in rat LMs which could be partly contributed by CYPs. The assessment of CYP isoforms would be helpful in identifying and understanding interspecies variation of YZG-331. In addition, the knowledge of species differences of YZG-331 mediated by CYPs will also help to extrapolate date obtained from animals to human reasonably well. However, till now, it was difficult to identify the isoforms mediated the metabolism in rats. It is well known that all main human individual human cDNA expressed CYPs are now commercially available (Martignoni et al., 2006). Nevertheless, for other species, the recombinant expressed individual CYPs are scarcely available.

Principal component analysis was further introduced to compare the variation in different species in YZG-331 conversion. The PC score plots showed specificity and dispersed clusters for different species (**Figure 12**), suggesting a characteristic interspecies difference for YZG-331 conversion. It is indicated that the CYPs polymorphism and enzymatic activity differences could cause variation in the metabolism of YZG-331. Therefore, it could increase the complexity when extrapolating data from animals to humans.

Our further research suggested that YZG-331 was metabolized by both FMOs and CYPs. It was well accepted that FMOs and CYPs share some substrates in common but often produce different metabolites (Guengerich, 2001). For most cases, the contribution of FMOs to drug metabolism might be underestimated, therefore, the number of drugs identified as being metabolized by FMOs is relatively small (Taniguchi-Takizawa et al., 2015). In contrast to CYPs, FMOs are not readily induced or inhibited by foreign chemicals (Phillips and Shephard, 2017). These differences between FMOs and CYPs indicate that drugs that are metabolized predominantly by FMOs would be less likely to elicit drug-drug interactions and potentially harmful side effects, and therefore the design of YZG-331 would offer clinical advantages. However, our results indicated that the metabolism of YZG-331 mediated by FMOs showed species variation (**Figures 10, 11**). In fact, various studies suggested that FMOs showed regional-specific expression and the species difference. There are distinct differences between the species in the metabolism of drugs *in vivo* by FMOs. For instance, drug substrates of FMO1 will be metabolized in mouse, but not human, liver; substrates of FMO2 will be metabolized in mouse, but not in the majority of humans (Phillips and Shephard, 2017). What is more, the relative contribution to overall activity of rat FMO1 in rat LMs was different from that of monkey and human FMO3. Recombinant expressed rat FMO1 had high affinity for the substrates with the exception of tozasertib (Yamazaki et al., 2014).

## Conclusion

Our results confirmed that YZG-331 could be metabolized by multiple pathways, including LMs and intestinal bacteria. CYP450 enzymes and FMOs mediated the metabolism of YZG-331, and the metabolic pathway showed species difference. Species variation in YZG-331 metabolism could affect the accuracy of the extrapolation from animals to humans.

## Ethics Statement

This study was carried out in accordance with the recommendations of Legislation Regarding the Use and Care of Laboratory Animals of China, and the protocol was approved by Animal Care and Use Committee of Peking Union Medical University.

## Author Contributions

LS and YL participated in research design; ZL, YY, and LS conducted the experiments and data analysis; ZL and LS wrote or contributed to the writing and revision of the manuscript.

## Conflict of Interest Statement

The authors declare that the research was conducted in the absence of any commercial or financial relationships that could be construed as a potential conflict of interest.
